# A Mouse Stromal Response to Tumor Invasion Predicts Prostate and Breast Cancer Patient Survival

**DOI:** 10.1371/journal.pone.0000032

**Published:** 2006-12-20

**Authors:** Marina Bacac, Paolo Provero, Nathalie Mayran, Jean-Christophe Stehle, Carlo Fusco, Ivan Stamenkovic

**Affiliations:** 1 Division of Experimental Pathology, Institut Universitaire de Pathologie Lausanne, Switzerland; 2 Department of Genetics, Biology and Biochemistry, University of Torino Torino, Italy; Ordway Research Institute Inc., United States of America

## Abstract

Primary and metastatic tumor growth induces host tissue responses that are believed to support tumor progression. Understanding the molecular changes within the tumor microenvironment during tumor progression may therefore be relevant not only for discovering potential therapeutic targets, but also for identifying putative molecular signatures that may improve tumor classification and predict clinical outcome. To selectively address stromal gene expression changes during cancer progression, we performed cDNA microarray analysis of laser-microdissected stromal cells derived from prostate intraepithelial neoplasia (PIN) and invasive cancer in a multistage model of prostate carcinogenesis. Human orthologs of genes identified in the stromal reaction to tumor progression in this mouse model were observed to be expressed in several human cancers, and to cluster prostate and breast cancer patients into groups with statistically different clinical outcomes. Univariate Cox analysis showed that overexpression of these genes is associated with shorter survival and recurrence-free periods. Taken together, our observations provide evidence that the expression signature of the stromal response to tumor invasion in a mouse tumor model can be used to probe human cancer, and to provide a powerful prognostic indicator for some of the most frequent human malignancies.

## Introduction

Malignant tumors are complex cellular ensembles composed, in addition to tumor cells, of host tissue-derived fibroblasts, endothelial cells, smooth muscle cells, and leukocytes. Despite self-sufficiency in growth signal generation and resistance to a variety of growth inhibitory and apoptosis-inducing stimuli, tumor cells rely on support from the host tissue for survival, growth and dissemination. In addition to constituting a reservoir of growth factors, the host tissue stroma provides the means to generate oxygen supply by supporting angiogenesis, as well as a structural scaffold for tumor cell adherence and migration [1]–[4]. Tumor cells must therefore possess the ability to exploit these resources to their advantage.

Access to extracellular matrix (ECM)-sequestered growth factors, initiation of angiogenesis and degradation of collagen and various ECM glycoproteins that constitute a natural barrier to invasion require the activation of a complex proteolytic enzyme machinery that initiates and maintains ECM remodeling [5], [6]. Numerous classes of extracellular proteinases are implicated in ECM remodeling including serine, aspartyl and cysteine proteases, members of the metzincin family, prominent among which are matrix metalloproteinases (MMPs), and adamalysin related proteinases [6]–[8]. Although some tumor cell types express a broad range of proteolytic enzymes that allow them to induce ECM remodeling by themselves, others lack the necessary proteolytic arsenal and must rely on enzymes supplied by stromal cells [9], [10]. By recruiting leukocytes, particularly macrophages, and by activating fibroblasts through growth factor secretion and cell-cell interaction, such tumor cells are believed to harness stromal cells into secreting MMPs and other proteases that promote ECM degradation and augment ECM-bound growth factor bioavailability.

Thorough understanding of host responses to different types of cancer growth, their prognostic significance and their potential value as therapeutic targets has been hampered in part by the approaches used to address them. Thus, tumor-host interactions and their consequences have been studied mostly in tumor cell-fibroblast co-culture systems and tumor xenograft models in immunocompromised mice where the stromal microenvironment may only partially reflect that of primary spontaneously arising tumors [11], [12]. Similarly, gene expression signatures of both primary [13], [14] and metastatic [15] tumors that may bear prognostic significance and predict metastatic proclivity, respectively, have for the most part been obtained from bulk tumor cell populations, such that the relative contribution of the tumor and stromal cell compartments could not be readily assessed.

To address the stromal response to tumor growth in a natural setting, and to assess its potential prognostic relevance, we examined the molecular events in the stromal cell compartment during cancer progression in a transgenic mouse model of multistage carcinogenesis. The choice of a mouse model rather than human tissues was based on experience from numerous studies that have highlighted the challenges associated with the use of archival human tissues, both from technical and biological viewpoints [16]. Variability as to sampling, tissue handling, processing and storing can all play a major role in obscuring potentially relevant gene expression profiles [16]. In addition, stromal responses to a given tumor may vary among patients according to patient age and coexistence of disorders unrelated to the malignancy. A well designed study to assess the stromal response to a human tumor should therefore be prospective and performed on a large number of individuals. While undoubtedly valuable, such an approach requires substantial time and should ideally be multicentric. Mouse tumor models, on the other hand, provide uniformity based on a defined oncogenic mechanism that drives tumor development, a unique genetic background and reduced inter-individual variability. Highly reproducible assessment of tumors at defined stages of evolution is therefore possible. Furthermore, late stage tumors free of therapeutic intervention are readily accessible in mouse models, in contrast to the corresponding patient tissues that are typically obtained following chemo- or radiation therapy.

The reproducibility of tumor development and progression in mouse models predicts reproducibility of the corresponding host tissue response and suggests that small numbers of animals may suffice to allow identification of relevant stromal response gene expression signatures. Such putative gene expression signatures can then be used to probe human cancers and the functional implication of the signature component genes in the disease process can be tested.

The neuroendocrine prostate tumors that arise in the mouse model used in the present study (CR2-TAg mice) have been previously characterized and shown to reproduce the stages of human tumor progression and metastasis [17], [18]. Microarray analysis of the stromal response to progression from intraepithelial to invasive tumors revealed a gene expression set consistent with ECM remodeling, characterized by the robust induction of genes encoding ECM proteins, growth factors, adhesion receptors and proteases. Remarkably, the gene expression set was found to have a powerful prognostic value in human prostate and breast cancer.

## Results

### Laser capture microdissection

**Figure 1:**
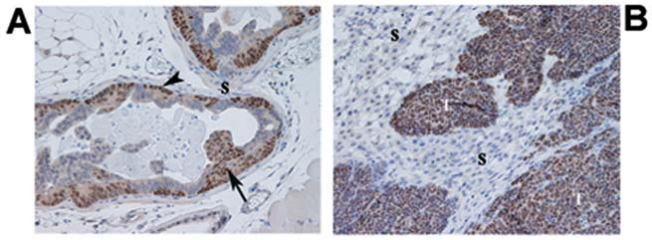
Histological appearance of PIN and invasive CR2-TAg prostate cancer lesions. (A) prostate glands of a 10-week old CR2-TAg mouse showing flat and tufted patterns of PIN (arrowhead and arrow, respectively), and a paucicellular stroma (S); (B) invasive cancer lesion from a 24-week old mouse where PIN acini have been replaced by solid tumor (T) and an abundant cellular, reactive stroma (S) composed primarily of fibroblasts/myofibroblasts as assessed by vimentin/actin smooth muscle staining (data not shown). Tissue sections were stained using anti-SV40 antibody (brown) and counterstained with haematoxylin. Magnification 100×.

Neuroendocrine prostate tumors that arise in CR2-TAg mice and evolve through a series of stages closely mimicking those observed in human prostate cancer have been previously described [17], [18]. Briefly, the mice are born with a normal prostate and develop prostate intraepithelial neoplasia (PIN) by 8 weeks, which progress to invasive carcinoma by 16–20 weeks, forming liver, lung, bone and lymph node metastasis by 24 weeks of age. The invasive stage is accompanied by a robust, predominantly fibroblastic, stromal reaction rendering the model attractive for addressing host tissue responses to invasive tumor growth. In the present study, prostates bearing early PIN lesions and invasive prostate tumors were removed from 10- and 24-week old CR2-TAg mice, respectively, at autopsy (Figure 1A, B). The choice of the 10 week time point was dictated by the observation that early invasion may already be present at 12 weeks [17]. By contrast, PIN lesions without evidence of microscopic invasion were abundant at 10 weeks whereas at 24 weeks, 100% of the mice displayed invasive cancer growth along with metastatic lesions. Following histological assessment of the tissues, the stromal compartment from both PIN lesions and invasive prostate tumors was selectively removed by LCM (Figure S1) and the RNA was extracted, amplified and subjected to microarray analysis.

### cDNA microarray analysis of microdissected stroma

A global gene expression profile of microdissected stroma was obtained on prostate tissue from 10 mice (4 with PIN, and 6 with invasive tumors). Gene expression analysis revealed 396 transcripts with differential expression between the two tumor stages (with a false discovery rate (FDR) of 15%). Among these, 256 displayed higher and 140 lower expression in invasive cancer stroma than in PIN-associated stroma (Table S1). Functional gene ontology (GO) annotation analysis revealed that one of the most significantly over represented gene families in the invasive tumor stroma was annotated to the term *endopeptidase activity* and contained transcripts encoding proteolytic enzymes, including lysosomal proteases, asparaginyl endopeptidases, matrix metalloproteinases and proprotein convertases (Table S2). Genes within this functional family that may be relevant to tumor progression encode cathepsins B, C, D, Z, legumain, a disintegrin-like and metalloprotease reprolysin type with thrombospondin type 1 motif 4 (*ADAMTS4*), matrix metalloproteinases 2 and 3 (*MMP2, MMP3*), and *FURIN*, which processes latent precursor proteins into their biologically active counterparts (Table 1).

**Table 1:**
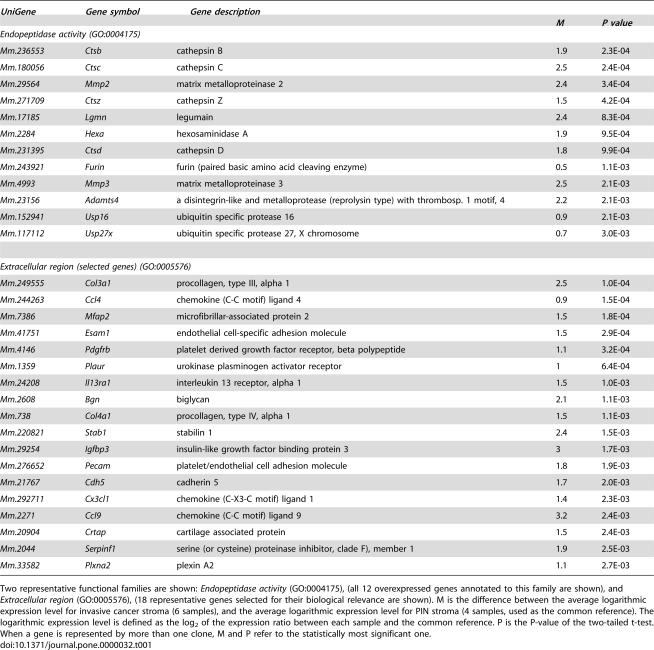
Selected genes found to be induced in invasive-cancer stroma when compared to PIN stroma.

*UniGene*	*Gene symbol*		*Gene description*		
				*M*	*P value*
*Endopeptidase activity (GO:0004175)*					
*Mm.236553*	*Ctsb*	cathepsin B		1.9	2.3E-04
*Mm.180056*	*Ctsc*	cathepsin C		2.5	2.4E-04
*Mm.29564*	*Mmp2*	matrix metalloproteinase 2		2.4	3.4E-04
*Mm.271709*	*Ctsz*	cathepsin Z		1.5	4.2E-04
*Mm.17185*	*Lgmn*	legumain		2.4	8.3E-04
*Mm.2284*	*Hexa*	hexosaminidase A		1.9	9.5E-04
*Mm.231395*	*Ctsd*	cathepsin D		1.8	9.9E-04
*Mm.243921*	*Furin*	furin (paired basic amino acid cleaving enzyme)		0.5	1.1E-03
*Mm.4993*	*Mmp3*	matrix metalloproteinase 3		2.5	2.1E-03
*Mm.23156*	*Adamts4*	a disintegrin-like and metalloprotease (reprolysin type) with thrombosp. 1 motif, 4		2.2	2.1E-03
*Mm.152941*	*Usp16*	ubiquitin specific protease 16		0.9	2.1E-03
*Mm.117112*	*Usp27x*	ubiquitin specific protease 27, X chromosome		0.7	3.0E-03
				
*Extracellular region (selected genes) (GO:0005576)*					
*Mm.249555*	*Col3a1*	procollagen, type III, alpha 1		2.5	1.0E-04
*Mm.244263*	*Ccl4*	chemokine (C-C motif) ligand 4		0.9	1.5E-04
*Mm.7386*	*Mfap2*	microfibrillar-associated protein 2		1.5	1.8E-04
*Mm.41751*	*Esam1*	endothelial cell-specific adhesion molecule		1.5	2.9E-04
*Mm.4146*	*Pdgfrb*	platelet derived growth factor receptor, beta polypeptide		1.1	3.2E-04
*Mm.1359*	*Plaur*	urokinase plasminogen activator receptor		1	6.4E-04
*Mm.24208*	*Il13ra1*	interleukin 13 receptor, alpha 1		1.5	1.0E-03
*Mm.2608*	*Bgn*	biglycan		2.1	1.1E-03
*Mm.738*	*Col4a1*	procollagen, type IV, alpha 1		1.5	1.1E-03
*Mm.220821*	*Stab1*	stabilin 1		2.4	1.5E-03
*Mm.29254*	*Igfbp3*	insulin-like growth factor binding protein 3		3	1.7E-03
*Mm.276652*	*Pecam*	platelet/endothelial cell adhesion molecule		1.8	1.9E-03
*Mm.21767*	*Cdh5*	cadherin 5		1.7	2.0E-03
*Mm.292711*	*Cx3cl1*	chemokine (C-X3-C motif) ligand 1		1.4	2.3E-03
*Mm.2271*	*Ccl9*	chemokine (C-C motif) ligand 9		3.2	2.4E-03
*Mm.20904*	*Crtap*	cartilage associated protein		1.5	2.4E-03
*Mm.2044*	*Serpinf1*	serine (or cysteine) proteinase inhibitor, clade F), member 1		1.9	2.5E-03
*Mm.33582*	*Plxna2*	plexin A2		1.1	2.7E-03

Two representative functional families are shown: *Endopeptidase activity* (GO:0004175), (all 12 overexpressed genes annotated to this family are shown), and *Extracellular region* (GO:0005576), (18 representative genes selected for their biological relevance are shown). M is the difference between the average logarithmic expression level for invasive cancer stroma (6 samples), and the average logarithmic expression level for PIN stroma (4 samples, used as the common reference). The logarithmic expression level is defined as the log_2_ of the expression ratio between each sample and the common reference. P is the P-value of the two-tailed t-test. When a gene is represented by more than one clone, M and P refer to the statistically most significant one.

Several of the other differentially expressed genes within the reactive stroma, annotated to the term *extracellular region*, were also candidate participants in the regulation of tumor growth and invasion. Thus, increased expression of genes encoding structural matrix components including, biglycan, procollagen type III, and IV, cartilage associated protein, regulators of insulin growth-factor bioavailability (IGFBP3), urokinase plasminogen activator receptor (PLAUR) and growth factor receptors (PDGFRB), may all play essential parts in the control of mesenchymal cell growth and differentiation, which may in turn influence tumor growth (Table 1).

### Validation of microarray results

**Figure 2:**
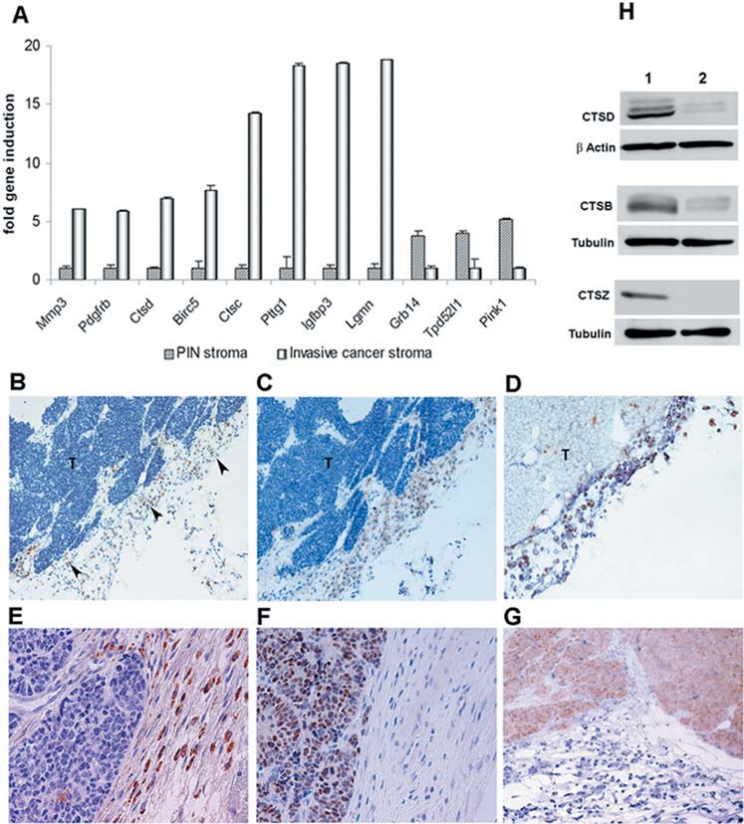
Validation of stromal genes identified by microarray analysis. (A) Quantitative real-time RT-PCR confirmed microarray results for 11 transcripts found to be differentially expressed between PIN and invasive cancer stroma. For a better representation, genes induced in the invasive cancer stroma were calibrated on the PIN stroma, those induced in the PIN stroma were calibrated on the invasive-cancer stroma. (B–G) Immunohistochemical validation of cathepsin D expression in invasive cancer stroma; cathepsin D (brown) was highly expressed in stromal cells (arrowheads) associated with invasive cancer, in contrast to tumor cells (T) where only occasional staining was seen (B,E); cells expressing cathepsin D were positive for vimentin (brown), confirming their mesenchymal origin (C); double staining of cathepsin D (brown) and vimentin (blue) highlighted their co-expression by fibroblasts/myofibroblasts (D); anti-SV40T antibody staining (nuclear, brown), (F), and double anti-cathepsin D/anti-SV40T antibody staining (blue/brown, respectively), (G), further confirmed that cathepsin D expression was primarily in stromal cells. Nuclei were counterstained with haematoxylin (B, C, E, F). Magnification 100× (B–D), 200× (E, G). (h) Western blot analysis confirmed increased expression of cathepsin D, B and Z in fibroblasts derived from CR2-TAg prostate cancers (1) compared to those derived from PIN prostates (2). Samples were collected from 24-week (invasive cancer) and 10-week old (PIN) mice, just as for the microarray experiments.

A subset of differentially expressed genes were validated by quantitative real-time RT-PCR on RNA extracted from microdissected PIN and invasive tumor stroma derived from animals that had not been used for microarray analysis. Genes selected for validation encode proteins implicated in proteolysis (matrix metalloproteinase 3 (MMP3), cathepsin C (CTSC), cathepsin D (CTSD), and legumain (LGMN)), modulation of insulin-like growth factor-1 bioavailability (IGFBP3), regulation of tumor and stromal cell growth, including platelet-derived growth factor receptor beta (PDGFRB), growth factor receptor bound protein 14 (GRB14), tumor protein D52-like 1 (TPD52L1) and PTEN-induced kinase 1 (PINK1), cell cycle regulation (pituitary tumor transforming gene, PTTG1), and survival (baculoviral IAP repeat-containing 5, BIRC5, Figure 2A). Consistent with the microarray data, *MMP3, PDGFRB, CTSD, BIRC5, CTSC, PTTG1, IGFBP3*, and *LGMN* were found to display, respectively, 6-, 6-, 7-, 8-, 14-, 18-, 19- and 19-fold higher expression in the invasive cancer stroma than in PIN stroma. In further support of the microarray data, GRB14, TPD52L1 and PINK1 displayed 4-, 4-, and 5- fold lower expression in invasive cancer than in PIN stroma (Figure 2A). Immunohistochemical analysis confirmed that expression of cathepsins D, B and Z was almost exclusively localised in the stroma of invasive tumors (Figure 2B, E and S2A, B) but was absent from PIN stroma (Figure S2C, arrowheads). Anti-vimentin antibody (Figure 2C), double anti-cathepsin D/anti-vimentin antibody (Figure 2D), and double anti-cathepsin D/anti-actin smooth-muscle antibody (data not shown) staining suggested that cathepsins were expressed predominantly by fibroblasts and myofibroblasts. Anti-SV40 antibody, which stained tumor cells only, and anti-cathepsin antibody staining patterns were mutually exclusive, indicating that cathepsin-positive cells within the stroma were not tumor cells that had detached and migrated away from the primary mass (Figure 2E–G). Western blot analysis of lysates from cultured fibroblasts obtained from prostates bearing PIN lesions and invasive cancer further confirmed the induction of cathepsins B, D and Z in invasive tumor-derived fibroblasts even after several days of culture (Figure 2H).

### Cross-species gene-expression comparison provides evidence that human orthologs of genes induced in the stroma of invasive CR-2TAg tumors predict prostate cancer patient survival

The gene expression profile of invasive tumor-associated stroma in the CR2-TAg mouse model is consistent with tissue remodeling and may conceivably reflect host tissue stromal response to some types of invasive cancer irrespective of species. To determine whether the mouse stromal genes identified herein are expressed in human prostate cancers and to test their potential relevance for patient survival, we developed a list of human orthologs of the mouse genes found to be differentially expressed between PIN- and invasive-cancer stroma. The genes within the list were subdivided into those that were upregulated (labeled “stroma up” genes) and those that were downregulated (labeled “stroma down” genes) in the invasive-cancer stroma (Table S3). A previously published data set of prostate cancer patients [13] for whom both gene expression and disease recurrence data were obtained was then analyzed for expression of the two groups of genes. Unsupervised hierarchical clustering was used to divide patients into two groups based only on the expression profiles of the genes in our lists. Standard statistical methods were used to determine (a) whether the two groups of patients thus defined showed statistically significant differences in terms of survival/recurrence-free time (Kaplan-Meier analysis), and (b) whether the genes within our lists had significantly higher predictive power than randomly selected genes (univariate cox analysis).

**Figure 3:**
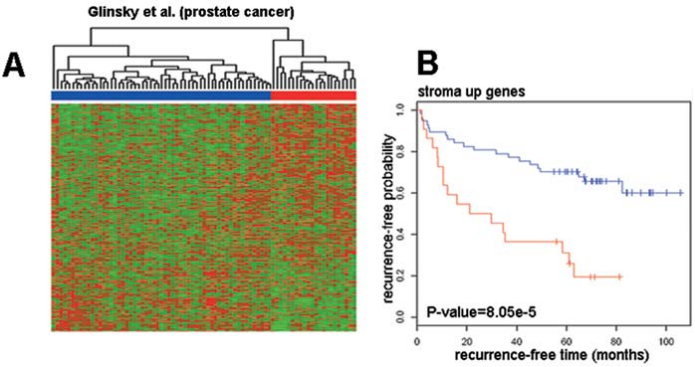
Prognostic value of “stroma up” genes for human prostate cancer. (A) Unsupervised hierarchical clustering of prostate cancer patients (columns) obtained using “stroma up” genes (rows). Red indicates high relative levels of gene expression and green represents low relative levels of gene expression. Genes in the cluster are ordered according to decreasing z values (Table S4). “Stroma up” genes divide prostate cancer patients in two main clusters (red and blue); (B) Kaplan-Meier survival analysis of the groups of patients defined by “stroma up” genes shows that the two groups of patients differ significantly in the overall survival time (p = 8.05×10^−5^; red, poor prognosis group; blue, good prognosis group). Similar analyses performed using “stroma down” genes can be found in Figure S3A.

Unsupervised hierarchical clustering of 79 prostate carcinoma patients [13] based on “stroma up” genes resulted in two patient groups (Figure 3A), with a significant difference in recurrence-free time as assessed by Kaplan-Meier survival analysis (log-rank test p = 8.05×10^−5^, Figure 3B). By contrast, groups of patients obtained based on “stroma down” gene clustering did not differ significantly in recurrence-free time (p = 0.086, Figure S3A). Cross-species gene-expression analysis therefore indicated that genes found to be induced in the stroma in response to tumor progression in CR2-TAg mouse model were not only expressed in human prostate cancer but were able to predict patient outcome.

### Survival-predictive ability of mouse stromal genes in different human cancers

Given that a stromal reaction to tumor invasion not only occurs in many cancer types but probably promotes tumor invasion and metastasis, it is conceivable that at least some of the molecular changes which occur in the tumor microenvironment during tumor progression may be common to different cancer types. To address this hypothesis we tested the applicability as a prognostic indicator of the stromal gene expression set identified in the CR2-TAg mouse model to several human malignancies known to induce a robust stromal reaction, including breast, lung and gastric carcinoma. The gene expression set was also tested in renal cell carcinoma, a tumor with a weak stromal response, where it was not expected to have prognostic value.

**Figure 4:**
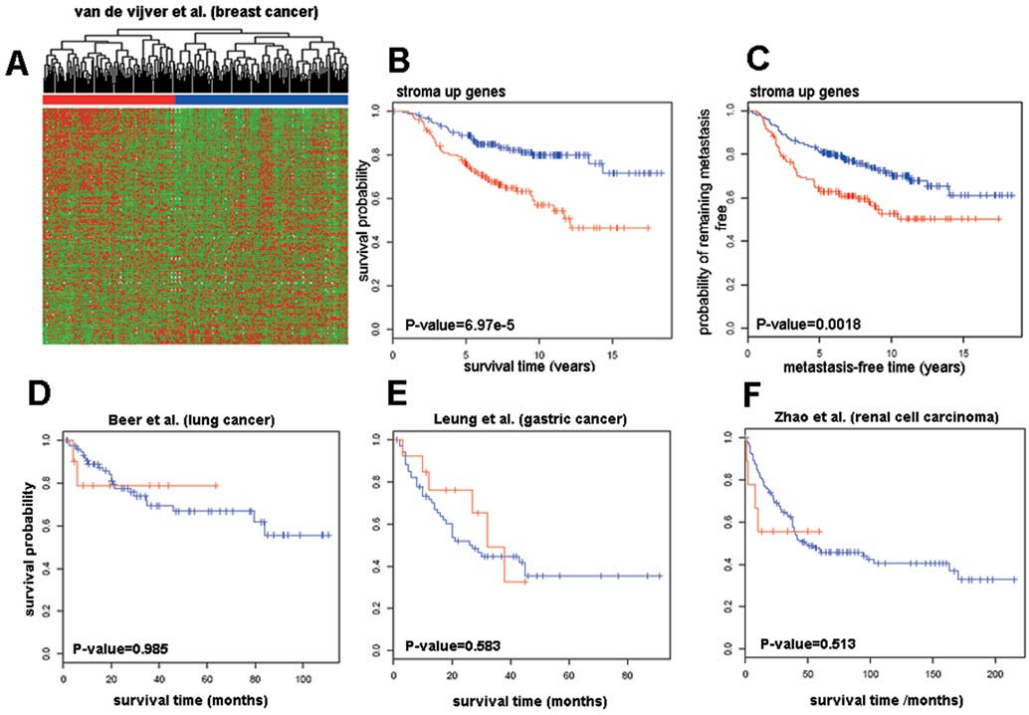
Prognostic value of “stroma up” genes in different human tumors. (A) Unsupervised hierarchical clustering of breast cancer patients (columns) obtained using “stroma up” genes (rows) ordered according to decreasing z values (Table S5). “Stroma up” genes divide breast cancer patients in two main clusters (red and blue). Kaplan-Meier survival analysis of the groups of patients defined by “stroma up” genes shows that the two groups of patients differ significantly in the (B) overall survival time (p = 6.97×10^−5^), and (C) metastasis-free time (p = 0.0018). Similar analyses performed using “stroma down” genes can be found in Figure S3B,C. (D–F) Kaplan-Meier survival analysis of lung (D), gastric (E), and renal cell carcinoma patients (F) shows that groups of patients defined by “stroma up” genes do not differ significantly in the overall survival time (p>0.05). Red, poor prognosis group; blue, good prognosis group.

Unsupervised hierarchical clustering of 295 early-stage breast carcinoma patients [14] using the list of upregulated stromal genes (“stroma up”) identified two groups of individuals (Figure 4A). Kaplan-Meier survival analysis of the two groups indicated that they were significantly different with respect to survival (p = 6.97×10^−5^, Figure 4B). The same analysis performed with respect to metastasis-free evolution instead of survival time showed that the two groups also differed significantly in the overall metastasis-free disease duration (p = 0.0018, Figure 4C). Consistent with our observations on prostate cancer, genes found to be downregulated in the stroma (“stroma down”) clustered the patients into groups that did not significantly differ in survival (p = 0.2, Figure S3B), or metastasis-free disease duration (p = 0.72, Figure S3C).

By contrast, when “stroma up” genes were used to cluster 86 primary lung adenocarcinomas (67 stage I and 19 stage III tumors) [19], the groups obtained did not display a significant survival difference (p = 0.985, Figure 4D). The same was true for a cohort of 90 primary gastric adenocarcinoma patients [20], (p = 0.583, Figure 4E). As expected, based on the paucity of the stromal response, unsupervised hierarchical clustering of 177 primary renal cell carcinoma patients [21], using “stroma up” genes failed to yield groups of patients with significantly different survival curves (p = 0.513, Figure 4F).

### Univariate cox analysis provides a list of genes related to survival

**Figure 5:**
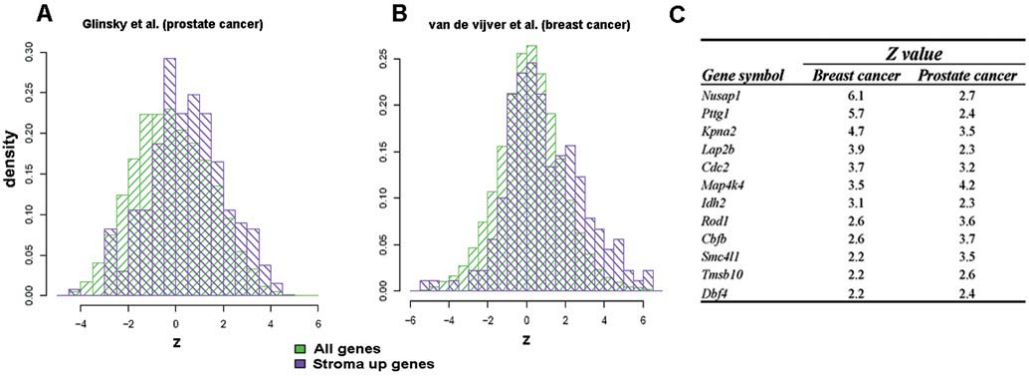
Univariate cox analysis. Representative histograms of (A) prostate and (B) breast cancer data sets obtained using univariate cox analysis of the correlation between the level of gene expression and survival time. Histograms show that the distribution of the z variable of “stroma up” genes (purple) is significantly higher than that of all genes present in the chip (green); (C) selected genes obtained by cross-list comparison of Tables S4 and S5 found to have strong predictive value for the survival of breast and prostate cancer patients. Only genes having a p value<0.05 in both tables were selected. The *z* value (and sign) indicate the strength of the correlation between the expression level of a gene and patient survival: the larger the positive value of *z* the greater the association of the overexpression of the corresponding gene with poor outcome.

Although unsupervised clustering and Kaplan-Meier analysis revealed that the overall list of “stroma up” genes had high prognostic value for prostate and breast cancer patients, it did not provide clues as to the identity of the genes that best predict survival. To address this issue, we performed univariate Cox analysis of the correlation between the level of gene expression and survival time. Such analysis produced, for each gene in our list, a z value indicating the strength and sign of the correlation: positive values of z>1.96 indicated that overexpression of the gene was statistically associated with poor prognosis (p<0.05), while negative values of z<−1.96 were statistically indicative of good prognosis (p<0.05), (Tables S4–8). In general, for prostate and breast data sets, “stroma up” genes tended to have higher z values when compared to all genes present in the chip, confirming that overexpression of these genes was associated with poor patient survival (schematically represented in Figure 5A, B).

A cross-list comparison of transcripts with statistically significant z values (p<0.05) in prostate and breast data sets (Tables S4, S5) identified 12 genes that were common to both lists. Among these genes were transcripts that encode transcription factors (CBFB), nuclear proteins that regulate nuclear import (KPNA2), proteins implicated in the structural organisation of the nucleus (TMPO), proteins involved in chromosome organization (PTTG1, SMC4l1), and a protein associated with centrosome separation (NUSAP1). Other genes whose overexpression was associated with poor survival of breast and prostate cancer patients encode regulators of differentiation (ROD1), protein kinases (MAPK4), and regulators of cytoskeletal organisation (TMSB10, Figure 5C). In addition to the 12 gene set, some of the “stroma up” genes displayed a tumor-specific association with prognosis. Thus, overexpression of genes including CX3CL1, FURIN and MTLG was associated with poor prognosis in breast cancer patients but a favourable outcome in prostate cancer patients. Similarly, high expression of the cathepsin family of proteases (CTSC, CTSZ, CTSB) was found to be indicative of poor prognosis in breast cancer patients but appeared to have no significant predictive value in prostate cancer patients. Univariate cox analysis thus confirmed that overexpression of “stroma up” genes was associated with poor patient outcome and allowed the identification of 12 genes that have strong predictive value for survival of prostate and breast cancer patients.

### Expression of PTTG1 and CTSD in human cancers

Two genes from the “stroma up” list were selected for immunohistochemical validation in human tumors, the primary goal being to determine their localisation rather than a precise assessment of their expression level. The securin gene (*PTTG1*) was selected because it displayed the strongest correlation with poor outcome of both prostate and breast cancer patients. The cathepsin D gene (*CTSD*) was selected because it was found to be (together with other members of the cathepsin protease family) one of the most strongly induced transcripts in the reactive stroma of the CR2-TAg mouse model and because its stromal expression has been associated with poor prognosis in breast cancer [22].

**Figure 6:**
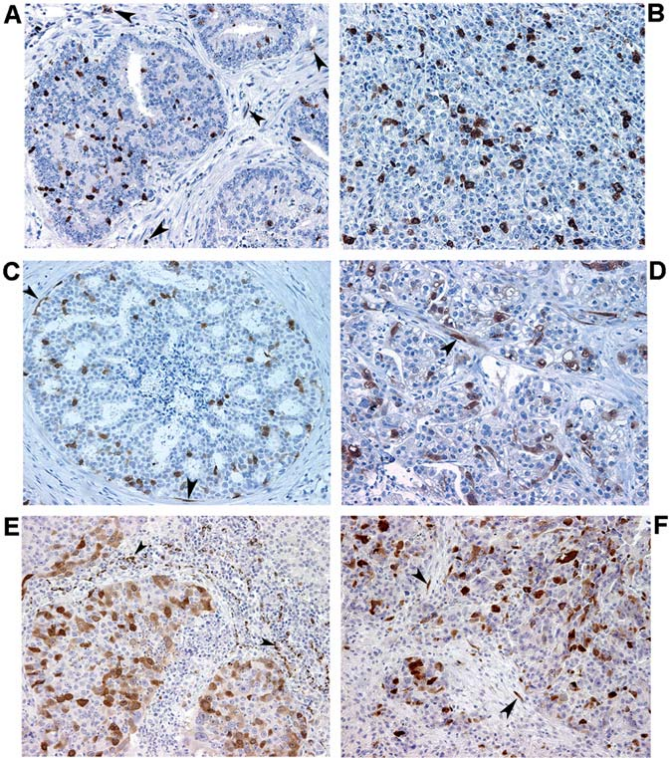
Histological pattern of securin expression in human cancers. Representative images of securin expression in human cancer samples. Securin expression (brown) is observed in tumor and stromal (arrowheads) cells in (A) late-stage PIN and (C) breast carcinoma *in situ* tissue sections. Invasive cancer stages of (B) prostate, (D) breast, (E) lung, and (F) ovarian cancers show strong securin expression by both tumor and stromal (arrowheads) cells. Nuclei were counterstained with haematoxylin. Magnification 200×.

Securin and cathepsin D expression were assessed by immunohistochemistry in an independent set of 20 prostate, 47 breast, 20 lung, and 11 ovarian cancer samples. Weak perinuclear securin expression was observed in occasional cells in normal prostate and breast epithelium but not in the corresponding stroma (data not shown). An increase in securin expression was observed in both epithelial and stromal cell compartments of late-stage PIN lesions and in breast carcinoma *in situ* (Figure 6A, C). A further increase in staining intensity along with redistribution to both cytoplasm and nucleus were observed in invasive cancer stages (Figure 6B, D), with the highest levels of expression observed in metastatic breast cancer lesions irrespective of location (data not shown). Elevated expression of securin was observed in all malignancies tested, including lung (Figure 6E) and ovarian cancer (Figure 6F). Consistent with the microarray data, securin expression was found to be increased in the stroma of all tumors analyzed compared to normal tissue stroma. Stromal fibroblasts and tumor-infiltrating leukocytes were among the stromal cells that expressed securin (Figure 6A–F, arrowheads).

**Figure 7:**
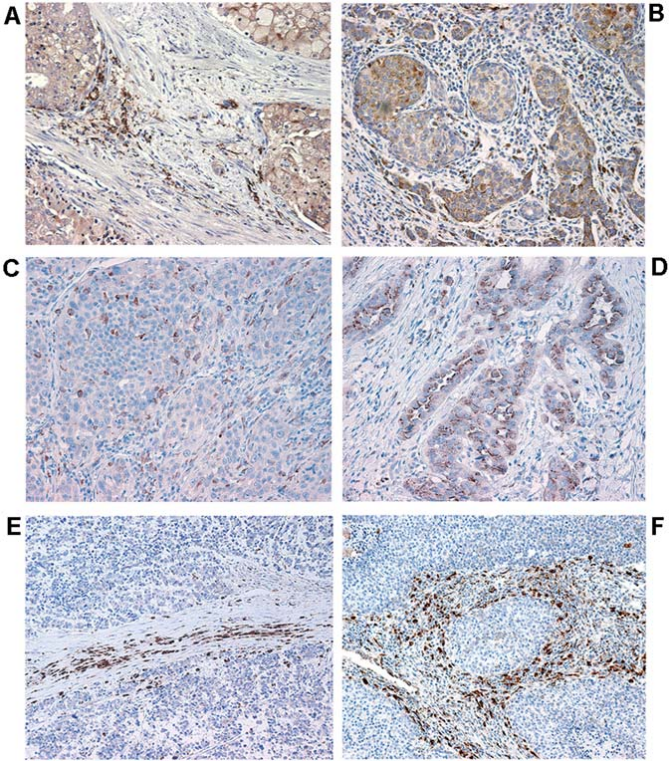
Histological pattern of cathepsin D expression in human cancers. Representative images of cathepsin D expression in human cancer samples. (A) prostate (B) breast cancer showing cathepsin D expression (brown) by tumor and stromal cells; (C) large-cell lung carcinoma and (D) lung adenocarcinoma showing cathepsin D expression by tumor cells and to a lesser extent by stromal cells; (E, F), cathepsin D is almost exclusively expressed by stromal cells in small-cell lung carcinomas (E) and large-cell neuroendocrine carcinomas (F), whereas tumor cells are almost devoid of cathepin D expression. Nuclei were counterstained with haematoxylin. Magnification 200×.

Cathepsin D expression was observed in both epithelial and stromal cells of prostate and breast tumors (Figures 7A, B), whereas normal tissues were largely devoid of anti-cathepsin D antibody staining (data not shown). Large cell lung carcinomas and lung adenocarcinomas displayed predominantly tumor-cell cathepsin D expression and weak stromal expression (Figure 7C, D). Remarkably, small cell lung carcinoma (SCLC) and large cell neuroendocrine lung carcinoma (LCNEC) displayed distinctive cathepsin D expression that was limited to the stromal cell compartment with little or no tumor cell staining (Figure 7E, F). This expression pattern was highly reminiscent of the one observed in the CR2-TAg mouse model.

### Stromal cathepsin D expression may influence tumor cell migration and proliferation

**Figure 8:**
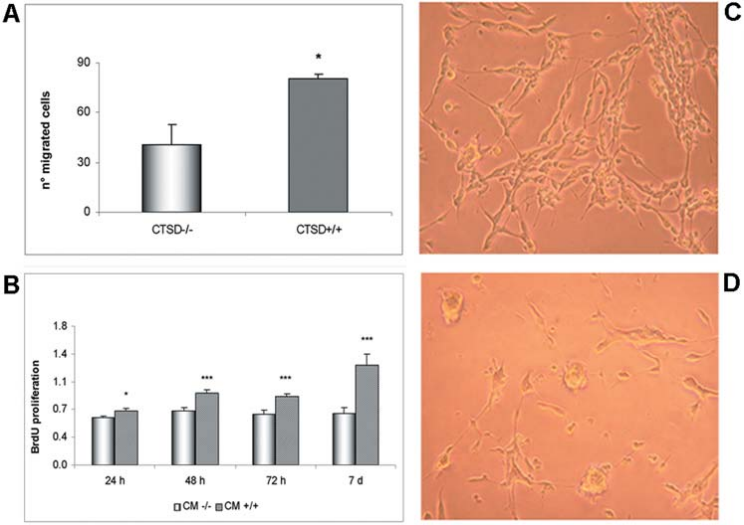
Stromal cathepsin D expression promotes PNEC cell migration and proliferation. (A) Quantification of PNEC cell migration in 3D matrigel co-culture with cathepsin D-deficient (CTSD^−/−^) or wild-type (CTSD^+/+^) fibroblasts after 30h of co-culture; (B) PNEC cell proliferation and (C, D) elongation are increased in the presence of conditioned medium (CM) derived from CTSD^+/+^ fibroblasts (C), compared to CM derived from CTSD^−/−^ fibroblasts (D). Experiments were performed three times, each time in quintuplicate. Representative results are shown. *p<0.05, ***p<0.001, Student t test.

Given that stromal cathepsin D expression is observed in a broad spectrum of tumors, we addressed its possible functional implications on tumor cell behavior. We first assessed migration of prostate neuroendocrine tumor cells derived form CR2-TAg mouse tumors (PNEC cells, [23]), in 3D matrigel co-cultures with wt (CTSD^+/+^) or cathepsin D-deficient (CTSD^−/−^) fibroblasts. PNEC cells displayed greater migration when co-cultured with CTSD^+/+^ than with CTSD^−/−^ fibroblasts (Figure 8A). Similar results were obtained using Boyden chamber assays where PNEC cells migrated as a function of the conditioned culture media derived from CTSD^+/+^ or CTSD^−/− ^fibroblasts used as the chemoattractant (data not shown). In addition, treatment of PNEC cells grown on plastic with conditioned media derived from CTSD^+/+^ fibroblasts resulted in increased proliferation (Figure 8B) and a phenotypic change characterized by an elongated neuronal-type morphology (Figure 8C). Conditioned culture medium from CTSD^−/−^ fibroblasts failed to induce these proliferative and morphological changes (Figure 8B, D). However, conditioned culture medium from CTSD−/− fibroblasts transfected with wt CTSD cDNA recapitulated the phenotypic changes of PNEC cells induced by wt fibroblast-derived medium (data not shown), suggesting that stromal cathepsin D expression may influence neuroendocrine tumor cell proliferation and motility.

## Discussion

The present work has identified a cross-species relevant stromal gene expression set in response to tumor invasion. Invasive CR2-TAg cancer stroma displayed induction of genes encoding numerous ECM proteins and ECM degrading enzymes, including *ADAMTS4, MMP2, MMP3 CTSB, CTSC, CTSD* and *PLAUR*. The combined substrate specificity of the induced proteolytic enzymes encompasses a broad range of ECM proteins, including collagens, fibronectin, fibrin, laminin and vitronectin, and latent growth factors, including among others, hepatocyte growth factor (HGF), TGF-β, and basic FGF [11], [24], [25]. The gene expression profile of stromal cells associated with tumor progression in CR2-TAg mice is therefore consistent with ECM remodeling functions that may be expected in a robust stromal reaction to processes ranging from mechanical tissue injury to tumor growth.

Stromal reaction to invasive tumor growth is widely believed to support tumor progression by providing growth factors, cytokines and ECM components that promote tumor cell survival, proliferation and migration [1], [3]. As such, at least some of the genes implicated in orchestrating stromal responses to tumor invasion should predict tumor evolution. Consistent with this notion, the gene expression signature identified in the present study bears a powerful prognostic value for human prostate and breast cancer, both of which are associated with a robust stromal response. However, its applicability to human cancers associated with a stromal reaction was not universal and could not predict the outcome of gastric and lung carcinoma. There are several possible explanations for this observation. First, a stromal response and the corresponding tissue remodeling are complex processes that comprise a broad range of cellular and molecular events and that may differ among tumor types, partly because of tumor characteristics and partly because of variability in host tissue properties from one organ to another. Thus, the stromal reaction typically associated with prostate and particularly breast cancer is rich in fibroblasts, myofibroblasts and ECM proteins, resembling that observed in the CR2-TAg model. Cancers that generate ulcerating lesions, such as gastric carcinoma, on the other hand, may be expected to induce a stromal reaction with a more prominent inflammatory component, characterized by an abundant leukocytic infiltrate and angiogenesis. It appears likely that these two types of stromal reaction display different gene expression signatures, each one having potential prognostic relevance to the type of cancer it is associated with. Second, the use of different technical approaches, including different types of microarrays containing non-identical gene sets relevant to the stroma and corresponding annotations, may, at least in part account for the apparent absence of signature consistency between studies [16], [26]. Third, most human tumor profiling studies have been conducted on bulk tumor tissue, such that it is impossible to know the relative representation of the stromal component which may vary significantly among samples, obscuring the emergence of a consensus signature. Clearly, substantial work will be required to generate and compare gene expression profiles of host responses to different tumor types using standardized technical approaches. However, candidate stromal gene expression signatures that are relevant to tumor progression may already emerge by comparing results of studies such as ours to those that have addressed the potential relevance to cancer of stromal cell gene expression profiles in a variety of pathophysiological conditions, including proliferative fibroblastic disorders and injury-associated tissue repair.

A recent study identified a set of genes whose expression pattern distinguished two proliferative fibroblastic disorders from each other, solitary fibrous tumors (SFT) and desmoid-type fibromatoses (DTF), and demonstrated that expression of this gene set was able to define two groups of breast carcinoma patients that differed significantly in overall survival [27]. Cross-study comparison between our set of “stroma up” genes and the “DTF genes” revealed 15 common transcripts (Table S9). Among genes common to the two signatures are several ECM components and ECM degrading enzymes, including COL1A2, COL3A1, BGN, MPAF2 and CTSC. By contrast, comparison to the “SFT gene set” of the same study did not yield significant overlap, as was the case for comparison of the “stroma down” genes to both the DTF and SFT gene sets. These observations suggest that the “stroma up” genes are more likely to be found in DTFs, known to be locally more aggressive and to have a higher degree of recurrence than SFTs.

A study focusing on the gene expression program in cultured primary fibroblasts in response to serum, believed to reflect the functional role of fibroblasts in wound healing, found that the “wound-response signature” was coordinately regulated in many human tumors and was a powerful predictor of the clinical course in several carcinomas [28], [29]. However, the overlap between the gene sets of this study and ours was limited to 8 genes (Table S9). This could be attributed, at least in part, to differences in experimental design, since Chang et al. addressed the effect of serum on in vitro cultured fibroblasts whereas we analyzed the stromal reaction to tumor progression in vivo, which implicates the contribution of several other components of the tumor microenvironment, including leukocyte infiltration, ECM deposition, and angiogenesis.

A third study used serial analysis of gene expression (SAGE) to assess the expression profiles of the epithelial and stromal cell populations of malignant breast tissue [30]. Comparison of these transcripts with the ones identified in the present work revealed several genes that were shared between our study and their “myoepithelial cell, myofibroblasts” and “stroma” SAGE libraries, including ADFP, ANXA1, BGN, CCL4, COL1A2, COL3A1, CXCR4, LAPTM5, MMP2, MT2A, SERPINF1, TMSB10 and TRA1 (Table S9).

It is noteworthy that two of the genes that were found to predict poor survival of prostate and breast cancer patients in the present study (*PTTG1* and *COL1A2*), form part of a 17-gene signature associated with metastases [15]. Expression of the *PTTG1* gene was among the most significant components within the prognostic gene expression signature in prostate and breast carcinoma. Its expression in the tumor cell compartment of prostate and breast cancer samples and its increase with the degree of tumor progression were consistent with its implication in tumor development [31]. Surprisingly, whereas most studies have reported its expression to be confined to the tumor cell compartment, we observed securin expression in both the tumoral and stromal compartments of invasive cancers. Elucidation of the role of securin in the tumor stroma will be of interest. It is attractive to speculate, for example, that securin-positive stromal cells reflect an active state that may contribute to tumor progression.

Another unexpected observation of the present work was the robust induction of cathepsins B, C, D and Z, in the invasive CR2-TAg cancer stroma mimicking the almost exclusively stromal cathepsin D expression pattern in human SCLC and LCNEC. Cathepsins have recently been shown to be upregulated in multistage pancreatic islet cell tumor model where they contributed to invasive tumor growth [32], [33]. They have also been suggested to participate in the progression of a variety of human cancers [34], [35] and their expression in breast cancer stroma has been shown to correlate with poor prognosis [22]. These findings are consistent with our present observations and reports by others that cathepsin D has mitogenic properties in tumor cells and fibroblasts [36]. Although the functional importance of stromal-derived cathepsins in tumor progression has yet to be fully elucidated, the stroma is known to provide MMP-mediated proteolysis in a variety of tumor models and some human cancers [9]. It is conceivable that in at least some tumor types cathepsins may function in an analogous manner.

Recent work by others has shown that cancer genes identified in mouse models can be used to probe human malignancies and to identify genes implicated in human cancer development and progression [37]–[40]. Our observations demonstrate the feasibility of using a mouse tumor model to identify a stromal gene expression set associated with tumor progression that is not only present in human prostate and breast cancer but that can predict the outcome of both malignancies. Several of the genes within the set identified in the present study have been associated with poor prognosis, recurrence and metastatic proclivity of several human tumors and their precise role in promoting tumor progression can now be assessed.

## Materials and Methods

### Animals and sample collection

Mice hemizygous for the CR2-TAg transgene were maintained in a specific pathogen-free facility according to Swiss guidelines for animal experimentation (authorization #1477). Samples (PIN prostates and prostate tumors) were collected from transgenic mice (CR2-TAg) expressing simian virus 40 large T antigen (SV40 TAg) in prostatic neuroendocrine cells under regulatory elements from the cryptidin-2 gene (*Defcr2*), described earlier [17], [18]. For the present study, prostate bearing early PIN lesions and invasive tumors with lung and liver metastases were collected from 10- and 24-week old mice, respectively. Tissues were isolated in RNase free conditions, snap-frozen in liquid nitrogen and stored at −80°C until use.

### Laser capture microdissection

LCM slides of prostate tissue from 10- and 24-week old mice bearing PIN lesions (n = 4 mice) and invasive cancer (n = 6 mice), respectively, were prepared from serial 8-µm-thick frozen tissues sections placed on a polyvinyl nuclease free membrane (Molecular Machines&Industries, Glattbrugg, CH). Tissue sections were fixed in ethanol 70% (30 sec), stained with haematoxylin and eosin (15 sec each), dehydrated in graded ethanol, treated with xylene and air-dried in a sterile laminar flow hood. Slides were microdissected immediately following staining using a µCut Laser Microdissection system (Nikon Eclipse TE200). All steps and solutions were performed under RNase free conditions. Generally, 1000–5000 cells were microdissected for subsequent RNA extraction. Microdissected stromal regions were within 0–100 µm and 0–300 µm from the epithelial compartment in the PIN and invasive-cancer tissue sections, respectively. All samples were subjected to histological examination prior to microdissection.

### RNA extraction, amplification and microarray analysis

Total RNA was extracted immediately following microdissection using the PicoPure RNA isolation kit (Arcturus, Mountain View, CA, www.arctur.com), according to the manufacturer's instructions. RNA was quantified using NanoDrop spectrophotometer (NanoDrop Technologies, Wilmington, Delaware, USA), and the concentration ranged between 10–50 ng/sample. RNA quality was assessed using the RNA 6000 Pico Assay Kit (Agilent). Only good quality RNA was subjected to two rounds of linear amplification using the RiboAmp™ RNA Amplification kit (Arcturus), according to the manufacturer's instructions, and aRNA was quantified using RNA 6000 Nano Assay Kit (Agilent). Labeled cDNA was obtained by reverse transcription of 5 μg of aRNA and incorporation of Cy3-dCTP and Cy5-dCTP (Amersham Biosciences, Amersham, UK). Microarrays containing 17,000 spotted cDNA clones were obtained from the Lausanne DNA Array Facility (http://www.unil.ch/dafl) and expression analysis performed using the NIA 17k clone set ([41] http://intranet.isrec.isb-sib.ch/microarrays/arrays_users.html). Hybridization of labeled cDNA to microarrays was performed for 16 h at 64°C in a humidified chamber (Corning Costar, Cambridge, MA). Microarrays were imaged using the ScanArray 4000 scanner (Perkin Elmer, Foster City, CA); Cy3 and Cy5 fluorescence intensities were extracted using the ScanAlyze software (http://rana.lbl.gov/EisenSoftware.htm). Gene expression was quantified with the SMA package using print tip group lowess normalization without background subtraction [42], [43]. For each array and each clone log2 ratios (M values) and the average log2 intensities (A value) of Cy3 and Cy5 signals were thus obtained. Intensity values produced by the image analysis software ScanAlyze as well as normalized gene expression data for all slides are available at GEO, accession number GSE5945.

### Experimental design and statistical analysis

For the identification of differentially expressed genes between stroma microdissected from the PIN- and invasive-cancer stages, samples from 10 mice, 6 with invasive cancer and 4 with PIN, were analyzed. The common reference (control) for all 10 samples was provided by pooled mRNA from the 4 PIN samples. In each of the 10 microarrays the control RNA (pooled from 4 PIN samples) was labeled with Cy3 and the test RNA (derived from each PIN lesion and invasive carcinoma) with Cy5. Log-ratios of the mRNA abundance for each clone were analyzed with a standard two-tailed, two-sample t-test to identify differentially expressed clones. To control the rate of false positives while taking into account the issue of multiple testing we used the Benjamini-Hochberg [44] method to produce lists of differentially expressed clones with a controlled false discovery rate (FDR).

### Functional analysis of differentially expressed genes

The lists of differentially expressed clones obtained as described above were analyzed from the point of view of functional annotation by searching for statistically overrepresented Gene Ontology (GO) annotations [45]. This analysis was performed separately for induced and repressed clones. Since GO terms are attributed to genes rather than to clones, the lists of differentially expressed clones were first translated into lists of genes (identified by Ensembl gene IDs [46]) using the Refseq ID, the gene symbols reported for each clone in the annotation of the microarray, and the correspondence between Refseq IDs, gene symbols and Ensembl IDs given by the Ensmart tool for browsing Ensembl. The lists of genes were in general shorter than the original lists of clones both because multiple clones were associated with the same gene and because clones that could not be associated with any gene were present. Gene Ontology annotations for all the genes associated with at least one clone spotted on the microarray were downloaded using ENSMART [47]. We then tested the lists of induced and repressed genes for overrepresentation of GO terms by applying the exact Fisher's test.

### Survival analysis of publicly available data

Gene expression and survival data for a cohort of prostate cancer patients was kindly provided by William Gerald [13]. Publicly available gene expression data for cohorts of breast cancer [14], lung cancer [19], gastric cancer [20], and renal cell carcinoma (RCC) [21] patients were obtained on-line together with corresponding survival data. When raw Affymetrix data were available (prostate and lung cancer) we applied the RMA algorithm {rma} to obtain gene expression data. In the other cases (breast, gastric cancer and renal cell carcinoma) we used the expression data provided by the authors. Unsupervised hierarchical clustering of the patients was performed using Pearson correlation coefficient to define dissimilarity between patient expression profiles, obtaining two clusters of patients in each case. The statistical significance of differences in survival probability between the two clusters was computed with the log-rank test. Univariate Cox analysis was performed to determine significant correlations between the expression profile of each individual gene represented on the chips and survival time. These analyses were performed using R {r} and the Bioconductor suite {biocond}.

### Real-time quantitative reverse transcription-PCR

cDNA was obtained using an Moloney murine leukemia virus reverse transcriptase and RNase H minus (Promega, Madison, WI). 50 ng of template total RNA were used per reaction. Real-time PCR amplification was done using a Taqman Universal PCR mastermix in an ABI Prism 7700 instrument (Applied Biosystems, Foster City, CA). Relative quantitation of target, normalized with an endogenous control (18s rRNA and GAPDH) was done using a comparative (Ct) method according to the manufacturer's instructions. Assays-on-Demand probes and primer mix (Applied Biosystems) were commercially available for MMP3 (Mm00440295_m1), CTSD (Mm00515587_m1), CTSC (Mm00515580_m1), IGFBP3 (Mm00515156_m1), eukaryotic 18S rRNA (Hs99999901_s1), and were designed using Primer Design program (Applied Biosystems) for PTTG1, BIRC5, PDGFRB, LGMN, GRB14, TPD52L1, PINK1. The sequences of the forward and reverse primers are provided in the supplemental materials and methods.

### Cell lines and proliferation assay

Prostate neuroendocrine cancer cells (PNEC) were previously described [23] and cultured in 75 cm^2^ Costar flasks coated with high molecular weight poly-L-lysine (0.1 mg/mL, P1274 Sigma), and laminin (2 µg/cm^2^, L2020, Sigma), in DMEM/F12 medium (Sigma) supplemented with 10% FBS, 1% non-essential amino acids (NEAA, Gibco), B27 serum supplement (17504-044, Invitrogen), 5 ng/ml EGF (354001, BDBiosciences) and 5 ng/ml bFGF (F5392, Sigma). Cathepsin D deficient (CTSD^−/−^) and wild-type fibroblasts (CTSD^+/+^) [36] were cultured in DMEM medium supplemented with 10% FBS and 1% NEAA. Conditioned medium (CM) was prepared using 80% confluent fibroblasts cultures in DMEM without FBS for 48 h. After collection, the CM was centrifuged at 800 g for 10 min, aliquoted and stored at −80°C if not immediately used.

For the proliferation assay, PNECs were trypsinized, counted and plated in 96-well plates (Costar) at 15 000 cells/100 µl medium/well. Medium was removed 24 h after plating, the cells washed once with PBS and incubated with the corresponding conditioned medium (100 µl/well) for 24–96 h. Cell proliferation was assessed using BrdU Cell proliferation ELISA assay kit (cat. 11 647 229 001, Roche) according to the manufacturer's instructions. Absorbance was measured using an ELISA plate reader at 410 nm with background subtraction at 492 nm.

### Immunohistochemistry

Paraffin-embedded tissue sections (4-µm thick) were deparaffinized and hydrated according to standard procedures. Endogenous peroxidase was quenched with 1% hydrogen peroxide for 15 min. Sections were subjected to antigen retrieval by boiling in citrate buffer (10 mM, pH 6.0) or EDTA (1 mM, pH 7.5) for 15 min, cooled, washed, and incubated with avidin/biotin blocking solutions to quench the endogenous biotin. Frozen 4-µm thick tissue sections were acetone-fixed and rehydrated prior to immunostaining and blocked for non-specific binding with 1% bovine serum albumin (Fluka). For single antibody staining, individual sections were incubated with primary antibodies (diluted as indicated below) for 40 min at room temperature (anti-SV40 and anti-securin antibodies were incubated overnight at 4°C). For double antibody staining, sections were incubated with the two antibodies in two serial steps. Sections were then processed using standard avidin–biotin immunohistochemical techniques according to the manufacturer's recommendations (Vector Laboratories, Burlingame, CA). Diaminobenzidine (DAB) was used as a chromogen for the single antibody staining, together with 5-bromo-4-chloro-3-indolyl phosphatase (BCIP/NBT) or Fast Blue for the double antibody staining.

The antibodies were purchased as follows: biotin conjugated anti-mouse Cathepsin D (BAF1029, 50 µg/ml, dil.1∶5), anti-mouse Cathepsin B (AF965, 100 µg/ml, dil.1∶10), and anti-Cathepsin X/Z/P (AF1033, 100 µg/ml, dil.1∶5), all from R&D Systems (San Diego, CA, USA); biotin conjugated anti-SV40 large T, small T antigen (cat. 554151, 0.1 mg, BD Pharmingen, dil. 1∶20); biotinylated anti-vimentin Ab-2 Clone V9 (MS-129-P1, 200 µg/ml, Neo Markers, Lab Vision Corporation, USA, dil.1∶50); anti-actin smooth muscle (Abcam Ltd, Cambridge, UK, dil.1∶50); mouse anti human securin (PTTG1) (ab3305, 0.2 mg/ml, Abcam, dil.1∶50); ECL streptavidin-HRP (from Amersham Biosciences); streptavidin-AP (Roche Applied Science); polyclonal rabbit anti-goat immunoglobulins/HRP, goat anti-rabbit immunoglobulins HRP, and BCIP/NBT substrate system (DakoCytomation); DAB tablets and Fast Blue BB salt (Sigma). For routine histopathological examination, 4-µm-thick frozen tissue sections were H&E stained according to standard procedures.

## Supporting Information

Figure S1. Example of laser-microdissected stroma. Example of laser-microdissected (LCM) stroma from tumor sections derived from 24-week old animals. (A) H&E-stained tissue section prior to LCM; (B) after LCM; (C) the remaining tissue section after the dissected stroma had been removed; (D) the microdissected stroma (measuring 300x1000μm). The sections were 8 μm thick, magnification 200x.
(1.69 MB TIF)

Figure S2. Immunohistochemical validation of cathepsin expression in CR2-TAg tissue sections. Immunohistochemical validation of (A) cathepsin B and (B) cathepsin Z expression (brown) showing almost exclusive stromal cell expression on sections derived from 24-week old CR2-TAg mice. Note that only occasional tumor cells within the same sections are stained for cathepsin B or Z; (C) cathepsin D (brown) is found to be expressed by epithelial, but not by stromal cells (arrowheads), on PIN sections derived from 10 week-old CR2-TAg mice. Nuclei were counterstained with haematoxylin. Magnification 200x.
(2.44 MB TIF)

Figure S3. "Stroma down" genes do not predict the survival of prostate and breast cancer patients. Kaplan-Meier survival analysis of (A) prostate and (B,C) breast cancer patients using "stroma down" genes. Note that the two groups of patients are not significantly different in the overall survival/recurrence-free and metastasis-free time (p>0.05).
(0.19 MB TIF)

Table S1. Differentially expressed clones identified by microarray analysis. List of differentially expressed clones between stroma microdissected from PIN and invasive-prostate cancers of CR2-TAg mice. The columns "M" and "STD M" contain mean and standard deviations of the log2 value of the expression ratio for the six invasive cancer samples; the next two columns refer to the four PIN samples. A pool of the four PIN samples was used as the reference for all 10 chips. The columns "t" and "P-value" give the statistical and the P-value for the two-sample, two-tailed t-test. M is the difference between the average logarithmic expression level for invasive cancer stroma (6 samples), and the average logarithmic expression level for PIN stroma (4 samples, used as the common reference). The logarithmic expression level is defined as the log2 of the expression ratio between each sample and the common reference.
(0.11 MB XLS)

Table S2. Functional Gene Ontology A list of ENSEMBL ids associated with the clones listed in Table S1 was generated using the ENSMART tool, which was also used to download the Gene Ontology annotation terms associated with each gene. The analysis was performed separately for "stroma up" and "stroma down" genes. "Genome_occurrences" indicates the number of genes represented in the chip and annotated to the GO term; "set_size" indicates the number of differentially expressed genes; "set_occurrences" indicates the number of genes annotated to the GO term among the differentially expressed ones; "expected_occurrences" indicates the number expected by chance alone; "p_value" indicates the P-value of Fisher's exact test; "cutoff" indicates the cutoff on such p-values derived from the simulation and corresponding to a 95% confidence level.
(0.09 MB XLS)

Table S3. Correspondence list between differentially expressed clones (0.10 MB XLS)

Table S4. Univariate cox analysis of clones used in the prostate cancer study. List of clones obtained using univariate cox analysis of "stroma up" and "stroma down" genes presented in Table S3, mapped to the prostate cancer data set [13]. The expression level of each gene was correlated with patient survival, resulting in the z value that indicates the strength of the correlation. Only genes having z values>1.96 or <-1.96, (p<0.05), were considered to be statistically significantly associated with patient survival. Positive values of z>1.96 indicate that overexpression of the gene is statistically associated with poor prognosis while negative values of z<-1.96 are statistically indicative of good prognosis. Genes are ordered according to decreasing z values.
(0.10 MB XLS)

Table S5. Univariate cox analysis of clones used in the breast cancer study. List of clones obtained using univariate cox analysis of "stroma up" and "stroma down" genes presented in Table S3, mapped to the breast cancer data set [14].
(0.08 MB XLS)

Table S6. Univariate cox analysis of clones used in the lung cancer study. List of clones obtained using univariate cox analysis of "stroma up" genes presented in Table S3, mapped to the lung cancer data set [19].
(0.05 MB XLS)

Table S7. Univariate cox analysis of clones used in the gastric cancer study. List of clones obtained using univariate cox analysis of "stroma up" genes presented in Table S3, mapped to the gastric cancer data set [20].
(0.09 MB XLS)

Table S8. Univariate cox analysis of clones used in the renal cell carcinoma study. List of clones obtained using univariate cox analysis of "stroma up" genes presented in Table S3, mapped to the renal cell carcinoma data set [21].
(0.09 MB XLS)

Table S9. List of common genes obtained by cross-study comparisons between our study ("stroma up" genes) and: "DTF genes" [27], the "wound-response signature" [28], and the "fibroblasts/myofibroblasts lists" [30].
(0.02 MB XLS)

Materials and Methods S1. Supplemental materials and methods. (0.03 MB DOC)
