# Supplementary figures and images for: A Mouse Stromal Response to Tumor Invasion Predicts Prostate and Breast Cancer Patient Survival
Source: PLoS One. 2006 Dec 20;1(1):e32. doi: 10.1371/journal.pone.0000032 (PMC1762322; doi:10.1371/journal.pone.0000032)

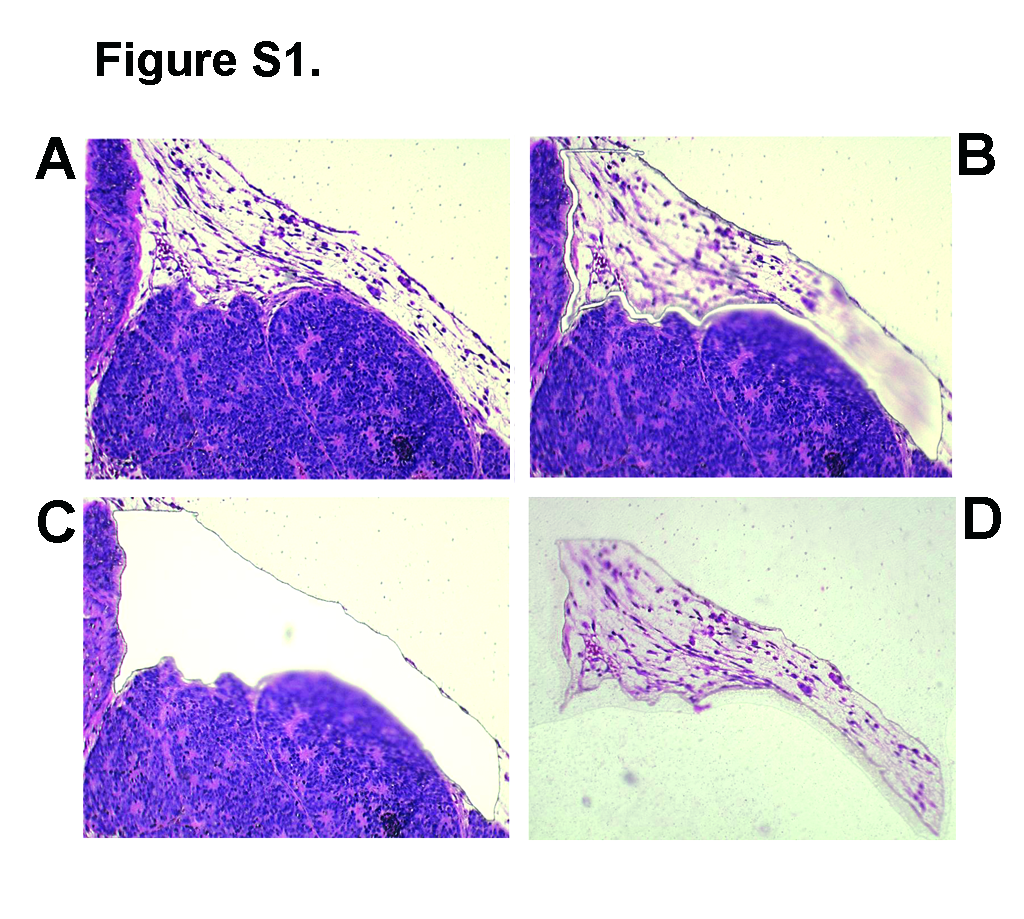

Supplement: Figure S1. Example of laser-microdissected stroma. — Example of laser-microdissected (LCM) stroma from tumor sections derived from 24-week old animals. (A) H&E-stained tissue section prior to LCM; (B) after LCM; (C) the remaining tissue section after the dissected stroma had been removed; (D) the microdissected stroma (measuring 300x1000μm). The sections were 8 μm thick, magnification 200x. (1.69 MB TIF) [file pone.0000032.s001.tif]

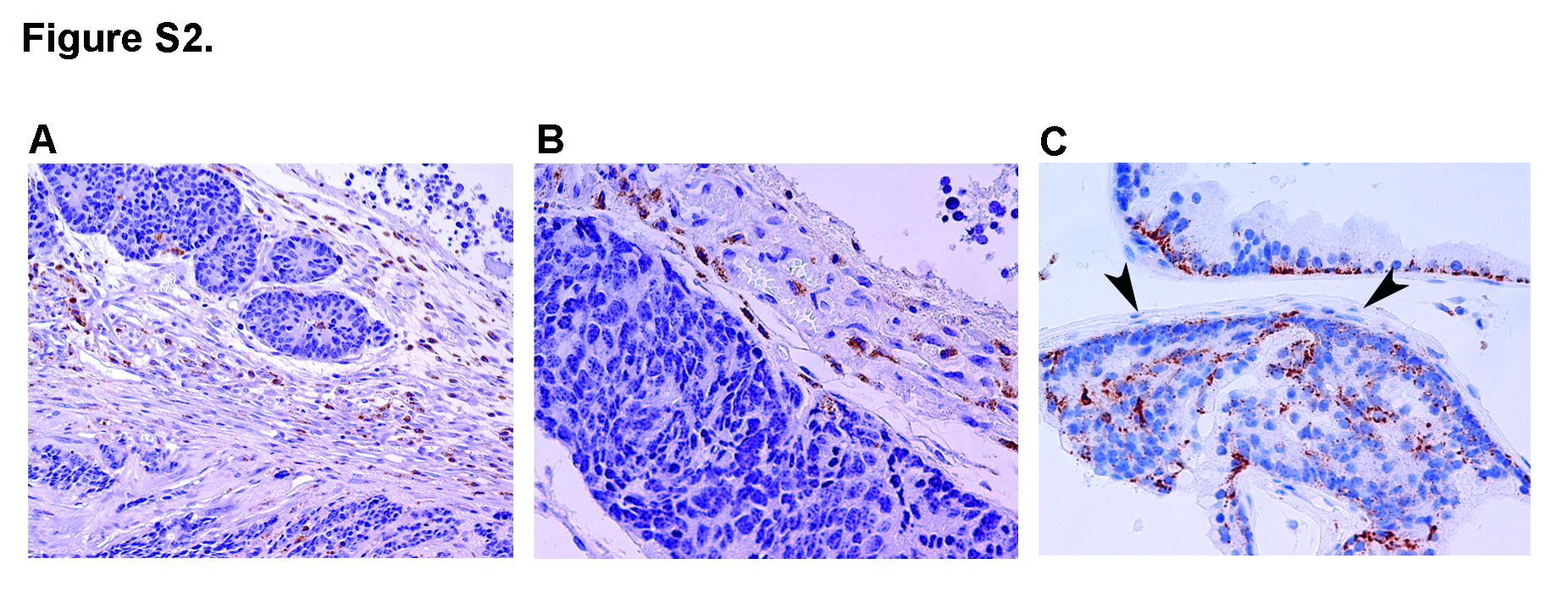

Supplement: Figure S2. Immunohistochemical validation of cathepsin expression in CR2-TAg tissue sections. — Immunohistochemical validation of (A) cathepsin B and (B) cathepsin Z expression (brown) showing almost exclusive stromal cell expression on sections derived from 24-week old CR2-TAg mice. Note that only occasional tumor cells within the same sections are stained for cathepsin B or Z; (C) cathepsin D (brown) is found to be expressed by epithelial, but not by stromal cells (arrowheads), on PIN sections derived from 10 week-old CR2-TAg mice. Nuclei were counterstained with haematoxylin. Magnification 200x. (2.44 MB TIF) [file pone.0000032.s002.tif]

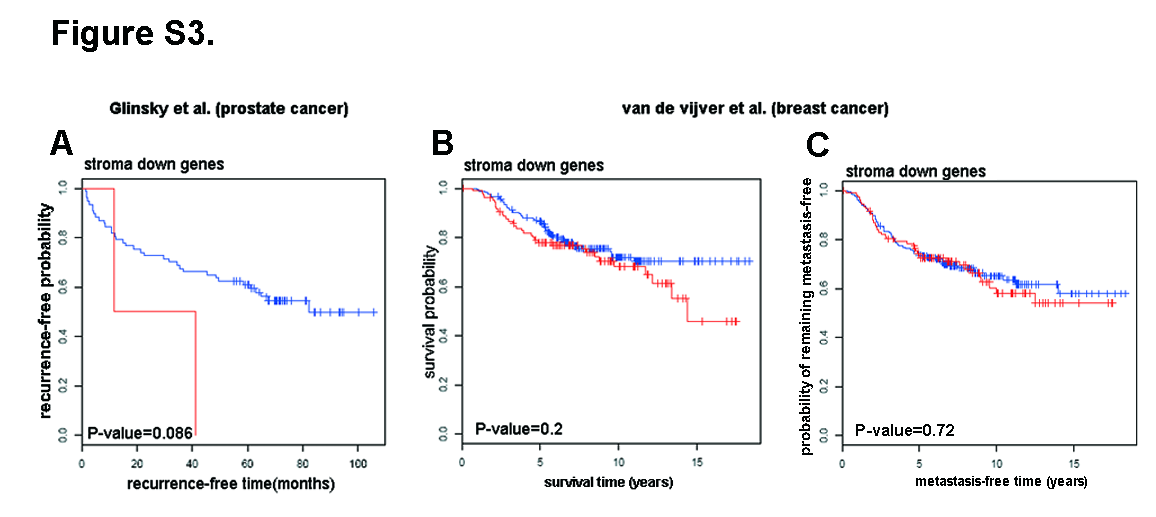

Supplement: Figure S3. "Stroma down" genes do not predict the survival of prostate and breast cancer patients. — Kaplan-Meier survival analysis of (A) prostate and (B,C) breast cancer patients using "stroma down" genes. Note that the two groups of patients are not significantly different in the overall survival/recurrence-free and metastasis-free time (p>0.05). (0.19 MB TIF) [file pone.0000032.s003.tif]
