# Supplementary material for: A Mouse Stromal Response to Tumor Invasion Predicts Prostate and Breast Cancer Patient Survival
Source: PLoS One. 2006 Dec 20;1(1):e32. doi: 10.1371/journal.pone.0000032 (PMC1762322; doi:10.1371/journal.pone.0000032)
Supplement: Materials and Methods S1. Supplemental materials and methods. — (0.03 MB DOC) [file pone.0000032.s013.doc]

**Supplemental materials and methods**

*Sequence of the forward and reverse primers used for quantitative real-time RT PCR*

**PTTG1** forward primer: 5’-AAGCCTATGAAGACTGGCAAACC-3’

reverse primer: 5’-GCAGGAACAGAGCTTTGTGTCTTA-3’

**BIRC5**  forward primer: 5’-AGATCTGGCAGCTGTACCTCAAG-3’

reverse primer: 5’- GGAGTGCTTTCTATGCTCCTCTTC-3’

**PDGFRB**  forward primer: 5’-AGCTCACGGTCTGAGCCATT-3’

reverse primer: 5’- GCTCGGACATTAAGGCTTGCT-3’

**LGMN**  forward primer: 5’-GCAAGCACTGGGTGGTGATT-3’

reverse primer: 5’- CCCGTTCCGGTGGATGA-3’

**GRB14**  forward primer: 5’-TCCCAGGGCCAATTCAAG-3’

reverse primer: 5’- CATCTCGGGCTGTGATGTCA-3’

**TPD52L1** forward primer: 5’-CTCGGCATGAATCTGATGAATG-3’

reverse primer: 5’- TGGCAGTTCCCACGTTATTG-3’

**PINK1**  forward primer: 5’-CCATCGCCTATGAAATCTTTGG-3’

reverse primer: 5’- GCAGCACATTTGCAGCTAAGC-3’

*Cloning, retrovirus generation and target cell infection*

A cDNA clone encoding the mouse cathepsin D was amplified using primers 5’-CCGCTCGAGCGGCCACCATGAAGACTCCCGGCGTCTTG-3’ (Fw) and 5’ GGAATTCCTTAGAGTACGACAGCATTG-3’ (Rev), from mouse fibroblasts by RT-PCR using Super Script one-step RT-PCR kit (Invitrogen, Carlsbad, CA) under the following conditions: one cycle at 50°C for 30 minutes and 94°C for 2 minutes followed by 35 cycles of 94°C for 45 seconds, 55°C for 45 seconds, and 72°C for 2 minutes and a final extension at 72°C for 10 minutes. The amplified fragment was digested with XhoI and EcoRI and inserted into the pMSCV puro resistance retroviral expression vectors (BD Biosciences Clontech, Palo Alto, CA). The plasmid was sequenced to verify the presence and integrity of cDNA encoding the mouse cathepsin D. Expression in CTSD^-/-^ fibroblasts was achieved using the Retroviral Gene Transfer and Expression (BD Biosciences Clontech), according to the manufacturer's instructions, and tested by western blot analysis using the biotinylated anti-mouse cathepsin D antibody as described below (San Diego, CA, USA). The infected cells were selected with 1 µg/mL puromycin for a maximum of 14 days, and bulk cultures of resistant cells were used.

*Isolation of fibroblasts and western blot analysis*

Fibroblasts were obtained from prostate tissue of four independent PIN and invasive-cancer bearing mice (10- and 24-weeks old, respectively). Tissues were isolated and manually processed under sterile conditions in PBS supplemented with 10% FBS, digested with collagenase VIII (0.1 mg/mL in PBS, Sigma) at 37°C with agitation for 40 minutes. The dissociated tissues were then incubated without shaking for 5 min at room temperature and supernatants filtered to a new tube and centrifuged for 6 minutes at 400 x g. The resulting cellular pellets were resuspended in DMEM containing 10% FBS, 1% P/S and cultured on tissue culture dishes. Confluent fibroblasts cultures were obtained 5 days after tissue dissociation and passaged for 2-3 population doublings within 14 days after tissue dissociation. Fibroblast purity was confirmed by immunofluorescence staining and positivity for vimentin and alpha-smooth muscle actin staining, and absence of SV40 positive tumor-cells and CD45 positive leukocytes (data not shown). Cell lysis, SDS-PAGE, blotting, and immunostaining were done according to standard procedures, and protein bands were detected using a chemiluminescent substrate kit (Amersham Biosciences, Amersham, United Kingdom), according to the manufacturer's recommendations. Biotinylated anti-mouse Cathepsin D (0.2 μg/ml), anti-mouse Cathepsin B (1 μg/ml) and Cathepsin X/Z/P (0.2 μg/ml), were from R&D Systems, secondary antibody was a rabbit-anti-goat horseradish peroxidase (HRP) conjugated (Bio-Rad, Hercules, CA).

*3D matrigel co-culture*

For 3D matrigel co-culture, PNECs and fibroblasts were pre-labeled with CellTracker Red (CMPTX, C-34552), and CellTracker Green (CMFDA, C-2925, both from Molecular probes) fluorescent dyes, respectively, according to the manufacturer’s instructions. 0.125x10^6^ of labelled PNECs were resuspended at 4°C in 0.3 ml of growth factor-depleted Matrigel (4.5 mg/ml, cat. 356231, BD Biosciences), added into one-half of the chamber (Lab-Tek®II Chamber #1.5 with borosilicate coverglass, Nalge Nunc International), and the Matrigel allowed to solidify for 45 min in an incubator at 37°C. 0.125x10^6^ of labelled fibroblasts (resuspended into growth factor-depleted Matrigel in the same way as PNECs) were then added to the second-half of the chamber and the Matrigel allowed to solidify for a further 45 min. The gels were then covered with DMEM containing 2% FBS and incubated for 30 h at 37°C. Prior to co-culture experiments, migration in 3D gels of the two cell populations was analysed separately and fibroblasts were identified as the non-migrating cell population. Images were acquired using laser scanning confocal microscopy (Zeiss LSM 510 Meta), and results obtained by 3D projection of all acquired scans (usually 80 scans/scanned area, 3 scanned areas/chamber). The total number of migrated PNECs (quantified by counting the red cells within the green fibroblast compartment) was normalized according to a formula where the z axis=1000 μm for all acquisitions. Independent experiments were performed three times, each condition in duplicate.
